# Vertical Technologies and Relational Values: Rethinking Ethics of Technology in an Age of Extractivism

**DOI:** 10.1007/s13347-025-00962-w

**Published:** 2025-08-30

**Authors:** Jeroen Hopster

**Affiliations:** Utrecht University, Utrecht, Netherlands

**Keywords:** Empirical turn, Vertical technologies, Socio-technical systems, Extractivism, Relational values, Intercultural philosophy of technology

## Abstract

Critical reflection on the material, environmental, and social conditions underlying technology remains peripheral to the field of technology ethics. In this commentary, I underwrite the diagnosis by Vandemeulebroucke et al. (2025) that the field suffers from an “extractivist blindspot”, but propose a somewhat different cure. First, rather than focusing on the material ontogenesis of technical artefacts, a more radical turn away from artefacts is called for, towards layered socio-technical systems as the field’s core object of analysis. Second, notwithstanding the merits of their intercultural proposal, I argue that in overcoming extractivism the conceptual resources of more adjacent philosophical traditions should not be overlooked.

## Introduction

Philosophy of technology has a blindspot. The ‘empirical turn’ it has witnessed, as exemplified in works by Ihde, Verbeek, Feenberg, and various others, has yielded a strong focus on concrete technological artifacts, to the neglect of their material constitution and environmental-social ontogenesis. This blindspot is criticized by Vandemeulebroucke et al. (2025), who argue that *extractivism* is the “unthought environmental-social condition of contemporary philosophy of technology.” Their critique applies with particular force to technology ethics, which “inadvertently fosters a techno-solutionist attitude”, whereas the political economy of extractivism remains unaddressed. Vandemeulebroucke et al. argue that the field needs an ontological shift. They draw on Māori philosophy to unlock new conceptual resources, which can illuminate how technological objects are materially and politically mediated.

Their general diagnosis of an “extractivist blindspot” is compelling, in particular in relation to empirical-turn-philosophy-of-technology. It is telling that environmental themes are almost entirely absent from contributions to the handbook *Philosophy of Technology after the Empirical Turn* (Franssen et al. eds. 2016). That said, there is room to push back against the authors’ identification of the empirical turn with contemporary philosophy of technology writ large. Debates about the empirical turn date back to the turn of the century. The field has expanded and diversified since and some recent contributions do exhibit a clear awareness of technology’s environmental constitution (e.g. Van Wynsberghe, 2021; Crawford, 2021). Recent monographs and handbooks devote at least one chapter to environment and sustainability (e.g., Floridi, 2023; Reijers et al., 2025).

Nevertheless, socio-environmental concerns remain peripheral and a more thoroughgoing ‘terrestrial turn’ is arguably needed (Lemmens et al., 2017), in particular to guide the field’s normative and critical aspirations. Consider the fact that there is hardly any discourse on the ethics of mining, a practice that dates back tens of thousands of years and has been integral to complex human societies, past and present (Sörlin, 2022). Or consider the notable divide between philosophy of technology and environmental philosophy. As already recognized in the 1990s, and highlighted in a recent volume on the topic (Kaplan ed. 2017, p.3), “environmental ethics overemphasizes wilderness and views human technological activity negatively, [whereas] philosophy of technology displays a ‘naïve anthropocentrism’ by focusing on the role of devices and machines on social, political, and economic affairs to the exclusion of ecological concerns.” Turning the gaze towards technologies’ material, environmental and social constitution is a much-needed corrective.

I underwrite this general diagnosis but propose a somewhat different cure in response. My first proposal is a more radical break with the field’s focus on *artifacts*, by considering *socio-technical systems* as the core object of analysis, cognizant of the political economy in which these systems are embedded. I will suggest that this approach can be furthered by a layered or ‘vertical’ understanding of socio-technical systems, which explicitly recognizes a ‘resource’ or ‘terrestrial’ layer as a core constituent (cf. Bratton, 2016; Sheikh, 2022).

My second proposal is to broaden the scope of traditions that can help the field to overcome its extractivist outlook. Indigenous epistemologies – non-extractive concepts developed by First Nations – contribute relevant resources, but they are not alone in doing so. Some more adjacent discourses are also well-equipped to provide philosophy of technology with a tailored, non-extractive conceptual repertoire. I will identify one concept – *relational values* – which arguably combines the best of both worlds: it is informed by indigenous ontologies, but amenable to uptake in scholarly philosophical discourse.

## Layered Socio-technical Systems

The kernel of Vandemeulebroucke et al.’s critique is that philosophers of technology treat concrete objects or artefacts as the starting point for theorizing, whereas the environmental-social conditions underlying them remain unquestioned. While the authors criticize this omission, they do not criticize the field’s focus on technological artefacts *as such*. In fact, they retain this focus, albeit with an explicit recognition of artefacts’ material, environmental, and social ontogenesis.

I believe a more radical departure from this artefactual focus is warranted, especially in the face of philosophy of technology’s environmental neglect. A key step to this effect is adopting *socio-technical systems* as the core object of analysis (Kudina & van de Poel, 2024). Some existing strands of theorizing are already prone to do so. For instance, socio-technical systems are the natural starting point for reflection on ‘socially disruptive technologies’ (Hopster, 2021), a label typically used to denote largescale technological systems (e.g. artificial intelligence, quantum technologies, or geo-engineering technologies), which are investigated in the context of institutions, governance, and conceptual changes.

Taking socio-technical systems as a starting point makes the socio-environmental conditions underlying contemporary technologies more perspicuous. However, neglect is still possible; the socio-technical systems approach, too, can benefit from a more thorough exploration of its underlying material conditions. Some moves in this direction can be discerned in recent work. An example is Crawford’s *Atlas of AI* (2021), which charts the economic, political, cultural and historical forces shaping AI, including the social practices, relations, institutions, global supply chains, as well as the extractive logic that make up the technology. Presenting AI as an ‘object’ or ‘artefact’ might serve to occlude these dimensions. Instead, AI is better understood as a complex socio-technical system with a layered composition, from terrestrial resources to satellites in outer space.

Recent work by Bratton (2016) and Sheikh (2022) provides a more explicit ontological underpinning of a layered understanding of socio-technical systems. Taking cue from the philosophy of Carl Schmitt as well as contemporary geopolitics, Sheikh and Bratton advance a ‘vertical’ understanding of these systems, consisting of different layers which engineers call *the stack* – the combination of parts required to create a digital product or service. Sheikh focuses on the stack of digital technologies, which consists of seven layers: resources, chips, networks, the cloud, intelligence, applications, and connected devices. For Sheikh’s purposes, this vertical understanding is relevant insofar as it is conducive to understanding the geopolitics of digital technologies. However, I submit that a vertical account could inform an understanding of socio-technical systems more broadly, and point to technology’s material constitution as an indispensable starting point for philosophical and ethical reflection. Such an approach holds promise for a socially and environmentally informed philosophy of technology, and more so than an ontology that retains technological artefacts as its core focus.

## Relational Values and Environmental Justice

On what traditions should the philosophy of technology draw to broaden its scope and overcome an extractivist outlook? Vandemeulebroucke et al. (2025) point to valuable conceptual resources from Māori philosophy. They argue that indigenous traditions are uniquely suited to provide such resources, given how distant they are from the extractivist paradigms that emerged in the Global North. Yet, they do not make a claim to exclusivity: insofar as their argument goes, contributions from other traditions can benefit the discourse as well. The following is meant as a friendly suggestion to complement their proposal.

Why would it be useful to seek further conceptual resources, beyond Māori philosophy? There are two concerns with limiting conceptual innovations to the latter tradition. The first concern is purely instrumental and has to do with considerations of conceptual uptake. Arguably, the extent to which new or ameliorated concepts can spread to a broader community of concept users is a key success criterion of conceptual engineering (Landes, 2025). It is my hunch that in this respect, advancing core Māori concepts such as *whakapapa* and *kaitiakitanga* might not be the most promising candidates for conceptual change in philosophy of technology, precisely because these concepts and their labels are so distant from, and unfamiliar to, the scholarly tradition. A limitation of the field to radically reinvent itself might be regarded as unfortunate, and perhaps my hunch that there is such a limitation is wrongheaded. But if it is not, and assuming that instrumental considerations are central to the field’s conceptual engineering ambitions,^1^ then drawing on distinctly Māori concepts might not be the most effective way forward.

Furthermore, there is an alternative, which retains essential contents of the proposal by Vandemeulebroucke et al., but does so in a language that has a greater affinity with the field and requires less contextual unpacking. As the authors explain, Māori ontology views local, time-bound relations as ontologically fundamental, thereby providing the conceptual tools for an alternative conceptualization of the relation between humans, living, and non-living nature. A very similar ontology informs the notion of *relational values*, which has recently been advanced by the Intergovernmental Science-Policy Platform on Biodiversity and Ecosystem Services, or IPBES (Pascual et al., 2023). Explicitely taking cue from First Nation epistemologies, as well as environmental scholars, IPBES proposes to regard the endorsement of relational values as a third mode of valuation, in addition to upholding intrinsic and instrumental values.

Consider a landscape or territory that is regarded as valuable because it is meaningful to the local population, whose identity is strongly connected with it. What underlies this valuing attitude can neither be understood in terms of instrumental value, nor in terms of intrinsic value. What makes the landscape valuable is grounded in the relational connection between humans and this specific, non-substitutable, natural environment (Neuteleers, 2020). The concept of relational value captures something that seems close to the combination of *whakapapa* and *kaitiakitanga* – unsurprisingly so, since Māori and other First Nation philosophies have informed the IPBES framework. Furthermore, a focus on relational value is more likely to receive wide scholarly uptake, given its close connection with other core concepts from environmental philosophy.

There are further resources philosophers of technology can draw on to overcome the field’s blindspot. Particularly relevant, in this regard, are resources from normative political philosophy, especially those that are anchored in ideals of recognition, participation, and restorative justice. Good examples are the discourse on environmental justice (Schlosberg, 2013) and climate reparations (Táíwò, 2022). These discourses are informed by the experiences of ecologically disadvantaged communities and the role of extractivism in perpetuating disadvantage. Philosophy of technology can draw on them to improve its conceptual apparatus.

I conclude that philosophy of technology indeed has an environmental blindspot. Vandemeulebroucke et al. (2025) provide a solid attempt at addressing this blindspot while broadening the canonical discourse, exemplifying the virtues of good intercultural philosophy of technology. The suggestions I have offered in this reply – to shift the ontological focus to layered socio-technical systems, and to draw on additional conceptual resources developed in more adjacent fields of philosophy and policy – are meant as constructive proposals to advance this project.

## Data Availability

No data were collected for this research.

## Footnotes

1 This instrumentalist assumption may be contested: there could be reasons of epistemic justice to favour the implementation of an ameliorative concept S over the implementation of a related ameliorative concept S*, even if S* has better prospects of uptake than S.
